# Lipoprotein Profiles in Class III Obese Caucasian and African American Women with Nonalcoholic Fatty Liver Disease

**DOI:** 10.1371/journal.pone.0142676

**Published:** 2015-11-23

**Authors:** Anna E. Garcia, Nader Kasim, Robyn A. Tamboli, Raul S. Gonzalez, Joseph Antoun, Emily A. Eckert, Pamela A. Marks-Shulman, Julia Dunn, Julia Wattacheril, Taylor Wallen, Naji N. Abumrad, Charles Robb Flynn

**Affiliations:** 1 Department of Surgery, Vanderbilt University Medical Center, Nashville, Tennessee, United States of America; 2 Le Bonheur Children’s Foundation Research Center, Memphis, Tennessee 38103, United States of America; 3 Department of Pathology, Vanderbilt University Medical Center, Nashville, Tennessee, United States of America; 4 Department of Medicine, Vanderbilt University School of Medicine, Nashville, Tennessee, United States of America; 5 Columbia University College of Physicians and Surgeons, New York, New York, United States of America; 6 Central Michigan University, College of Medicine, Mount Pleasant, Michigan, United States of America; University College London, UNITED KINGDOM

## Abstract

Triglyceride content in the liver is regulated by the uptake, production and elimination of lipoproteins, and derangements in these processes contribute to nonalcoholic fatty liver disease (NAFLD). Previous studies show a direct relationship between intrahepatic fat and production of apolipoprotein B100 (apoB100) containing particles, VLDL and LDL, but little consensus exists regarding changes in lipoprotein production in the development of simple steatosis (SS) versus nonalcoholic steatohepatitis (NASH). Further, ethnic variations in lipoproteins among SS and NASH are unknown as is how such variations might contribute to the differential prevalence of disease among Caucasians versus African Americans. In this study, we assessed plasma lipoprotein profiles by nuclear magnetic resonance (NMR) spectroscopy in 70 non-diabetic class III obese females recruited from the surgical weight loss clinic. Of these, 51 females were stratified by biopsy-staged NAFLD severity (histologically normal, SS, or NASH). NASH females displayed increased circulating triglycerides and increased VLDL particle number and size relative to those with histologically normal livers, while total and large LDL concentration decreased in SS versus NASH and correlated with increased insulin resistance (via HOMA2-IR). When Caucasian women were examined alone (n = 41), VLDL and triglycerides increased between normal and SS, while total LDL and apoB100 decreased between SS and NASH along with increased insulin resistance. Compared to Caucasians with SS, African American women with SS displayed reduced triglycerides, VLDL, and small LDL and a more favorable small to large HDL ratio despite having increased BMI and HOMA2-IR. These findings suggest that ApoB100 and lipoprotein subclass particle number and size can delineate steatosis from NASH in obese Caucasian females, but should be interpreted with caution in other ethnicities as African Americans with SS display relatively improved lipoprotein profiles. This may reflect variation in the relationship between dyslipidemia and NAFLD progression across gender and ethnicity.

## Introduction

Hepatic steatosis stems from an imbalance between lipid supply and demand where excess fatty acids are stored in the liver as fat [1]. Obese individuals in particular have surplus fatty acids and increased hepatic steatosis due to dietary intake and the tendency to become insulin resistant. With decreased insulin sensitivity, insulin can no longer inhibit lipolysis in peripheral tissues, resulting in more circulating free fatty acids for potential storage in the liver [2]. Once accumulated, hepatic triglyceride (TG) is eliminated from the liver via lipid oxidation or the secretion of TG-rich very-low density lipoproteins (VLDL) depending on local or peripheral energy needs [3, 4]. However, this lipid and lipoprotein balance can become perturbed in the setting of obesity and type 2 diabetes (T2D), and may contribute to the development of nonalcoholic fatty liver disease (NAFLD) [5].

NAFLD encompasses a spectrum of disease ranging from excess fat in more than 5% of hepatocytes (termed simple steatosis; SS) to nonalcoholic steatohepatitis (NASH), an advanced inflammatory phenotype hallmarked by hepatocyte ballooning and necrosis [6]. NAFLD, especially NASH, is associated with hepatic and global metabolic dysfunction, including increased insulin resistance [5]. While over 25% of the general population has NAFLD, this number jumps to 90% in class III obese subjects (BMI ≥ 40) and to 100% in those with T2D [7, 8]. The prevalence of the disease differs among ethnicities, with African Americans exhibiting a lower prevalence of steatosis and NASH relative to Caucasians and Hispanics [8, 9]. Elevated levels of TG, VLDL and small dense low-density lipoprotein (LDL) and decreased levels of high-density lipoprotein (HDL) are associated with the development of cardiovascular disease and diabetes. [3, 10]. Interestingly, African Americans exhibit lower levels of TG and higher levels of HDL for a given BMI and degree of insulin resistance [11]. However, it is unclear how lipoproteins vary in SS versus NASH of any ethnicity, with some studies reporting increased production of VLDL and LDL in NASH relative to SS while others report a decline [12–17].

In this study, we examined lipoprotein profile differences among class III obese females with histologically-determined SS or NASH and compare them to lipoprotein profiles from histologically normal-weight-matched female controls. We found distinct differences in lipoprotein and lipid profiles that may aid in noninvasively determining NAFLD diagnoses and severity progression [12, 17–19].

## Materials and Methods

### Subjects

The Internal Review Board of Vanderbilt University approved this study which is registered at ClinicalTrials.gov (NCT00983463). All studies met NIH and institutional guidelines for human subject research and the Declaration of Helsinki. All subjects gave informed written consent prior to participation. Caucasian and African American Class III obese females (BMI > 40 kg/m^2^) scheduled for bariatric surgery were recruited from the Vanderbilt Center for Surgical Weight Loss (Nashville, TN). Inclusion criteria included non-sedentary, non-diabetic women between the ages of 23 and 60 years. Screening included anthropomorphic measures, clinical history, physical exam, and laboratory tests including complete blood count with differential, liver function tests, and fasting blood glucose. Ethnicity was self-reported. Exclusion criteria included presence of viral hepatitis, autoimmune hepatitis, hemochromatosis, significant alcohol use (>14 drinks per week over a 2-year period), intercurrent infections or cancer, previous gastric, duodenal, proximal jejunum or pancreatic resection surgery, and positive serum pregnancy test. Lean females (BMI ≤ 27 kg/m^2^) were recruited from the Vanderbilt community and had no significant past medical history. Liver biopsies were not available for these women as it is discouraged to obtain non-clinically indicated liver biopsies due to the risks associated with the procedure. Further, we did not utilize AST and ALT to screen for liver disease in control patients given the values are not sensitive enough to screen for NAFLD nor are they a reliable indicative of liver disease severity [20–22].

### Blood and tissue sampling

Women were studied after an overnight fast (≥10 hours). Blood was collected from lean patients by the laboratory nurse practitioner in the morning following an overnight fast. Blood was collected from patients receiving bariatric surgery preoperatively prior to induction of anesthesia. The attending surgeon performed intra-operative wedge liver biopsies collected from the left lobe of the liver for these patients as well.

### Plasma analyses

Plasma glucose concentration was determined using an automated glucose analyzer (YSI 2300 STAT plus, Yellow Spring Instrument Co., Yellow Springs, OH, USA). Plasma insulin concentrations were measured by radioimmunoassay (Linco Research, St. Louis, MO, USA). Plasma insulin and glucose were used to calculate insulin resistance using the Homeostatic Model Assessment of Insulin Resistance with physiologic adjustments (HOMA2-IR) [23]. Hospital lab services assessed aspartate transaminase (AST) and alanine transaminase (ALT) in surgical patients via enzymatic methods, albumin via a bromo-cresol green albumin assay, and platelets via Sysmex Automated Hematology System (Sysmex America, Lincolnshire, IL. USA).

Lipoprotein subclass concentration and particle size were assessed via NMR (nuclear magnetic resonance) spectroscopy (Liposcience/LabCorp, Raleigh, NC, USA) using the Lipoprofile-3 algorithm. Concentrations (nanomoles/L) of total VLDL, LDL, HDL and the following subclasses were determined according to particle size (nanometers, nm): large VLDL (>60.0 nm), medium VLDL (42.0–60.0 nm), small VLDL (29.0–42.0 nm), IDL (23.0–29.0 nm), large LDL (20.5–23.0 nm), small LDL (18.0–21.2 nm), large HDL (9.4–14.0 nm), medium HDL (8.2–9.4 nm) and small HDL (7.3–8.2 nm) [24]. Weighted average particle size for VLDL, LDL, and HDL was calculated based on subclass concentration and associated diameters. NMR calculated lipid levels highly correlate with respective chemically measured levels of plasma triglyceride (r>0.9), HDL cholesterol (r>0.9), and LDL cholesterol (r = ~0.8–0.9) while providing the added benefit of quantifying particle concentrations and size [25]. Because each VLDL and LDL possesses one apolipoprotein B100 (apoB100) molecule, previous methods used a combination of centrifugation and apoB100 quantification assays to separate and quantify the lipid classes. NMR-derived levels strongly correlate with these methods (r = 0.928) as well as LDL and HDL levels determined by gradient gel electrophoresis (r = 0.946 and r = 0.953)[24].

In addition, we employed a novel means of describing insulin sensitivity. Classically, insulin resistance is defined by the degree of insulin’s effect on glucose metabolism, but several investigators have emphasized the importance of lipid abnormalities in the pathogenesis of insulin resistance [26, 27]. NMR studies suggest a more “lipocentric” view of insulin resistance after finding significant associations between specific lipoprotein profile constituents and insulin resistance [28, 29]. The 6 parameters most associated with insulin resistance include: increased number of individual particles of large VLDL and small LDL, decreased number of large HDL particles, bigger VLDL sizes and smaller LDL and HDL overall sizes. Shalaurova and Otvos et al. used this information to create an equation that can use lipoprotein profiles to describe insulin sensitivity based on each parameter’s individual association with insulin resistance. This Lipoprotein Insulin Resistance (LP-IR) Index or Score assigns each lipid parameter an individual weight as follows: VLDL size, 32; large VLDL, 22; HDL size, 20; large HDL, 12; small LDL, 8; and LDL size, 6. The sum of these weights creates the LP-IR score, which ranges from 1–100, with 100 being the most insulin resistant [30]. The scores were calculated by the Liposcience group during NMR analysis, and the algorithm used is described in detail in the manuscript by Shalaurova and Otvos et al. [30].

### Histopathologic Determination of NAFLD Severity

Diagnosis of normal, simple steatosis, or steatohepatitis pathology was made by retrospective chart review of pathology reports for the liver biopsies collected at the time of surgery by the attending. Women were diagnosed as having simple steatosis when fat was present in greater than 5% hepatocytes or NASH if the pathologist described the sample as “steatohepatitis” based on the degree of lobular inflammation and ballooning [31]. Of note, the current investigators were able to secure slides for 31 of the 51 women in the study in order to evaluate the biopsies via the NAFLD Activity SCORE (NAS) (Table A in S1 File) and confirm hepatopathologist findings with the help of an independent hepatopathologist. The NAS Score is a sum of independent scores for ballooning, lobular inflammation and steatosis [32]. Women with a NAS score of 0–1 were categorized as “normal,” 2–3 as “simple steatosis” (SS) 4 or above as “NASH”. Fibrosis was scored as an independent variable. Unfortunately, due to the retrospective nature of this study, slides were not available for all samples. Because of this, we ultimately elected to classify livers based on the original histopathological classification at the time of surgery that was recorded in the electronic medical record, as this classification was uniformly determined and available for all women.

### Statistics

Results for two groups were compared by unpaired, two-tailed Mann-Whitney test (t-test) using Prism 5 (Graphpad Software). Differences among three groups were analyzed by analysis of variance (ANOVA) when variables were normally distributed by the Kolmogorov-Smirnov test or upon log transformation. All data are presented as mean ± standard error (SEM). Statistical significance was established at *P*-value ≤ 0.05. Regression analyses (Table B in S1 File) were used to estimate linear relationships among variables after log transformation of skewed data.

## Results

### Anthropometric Measurements in Class III Obese

**Fig 1:**
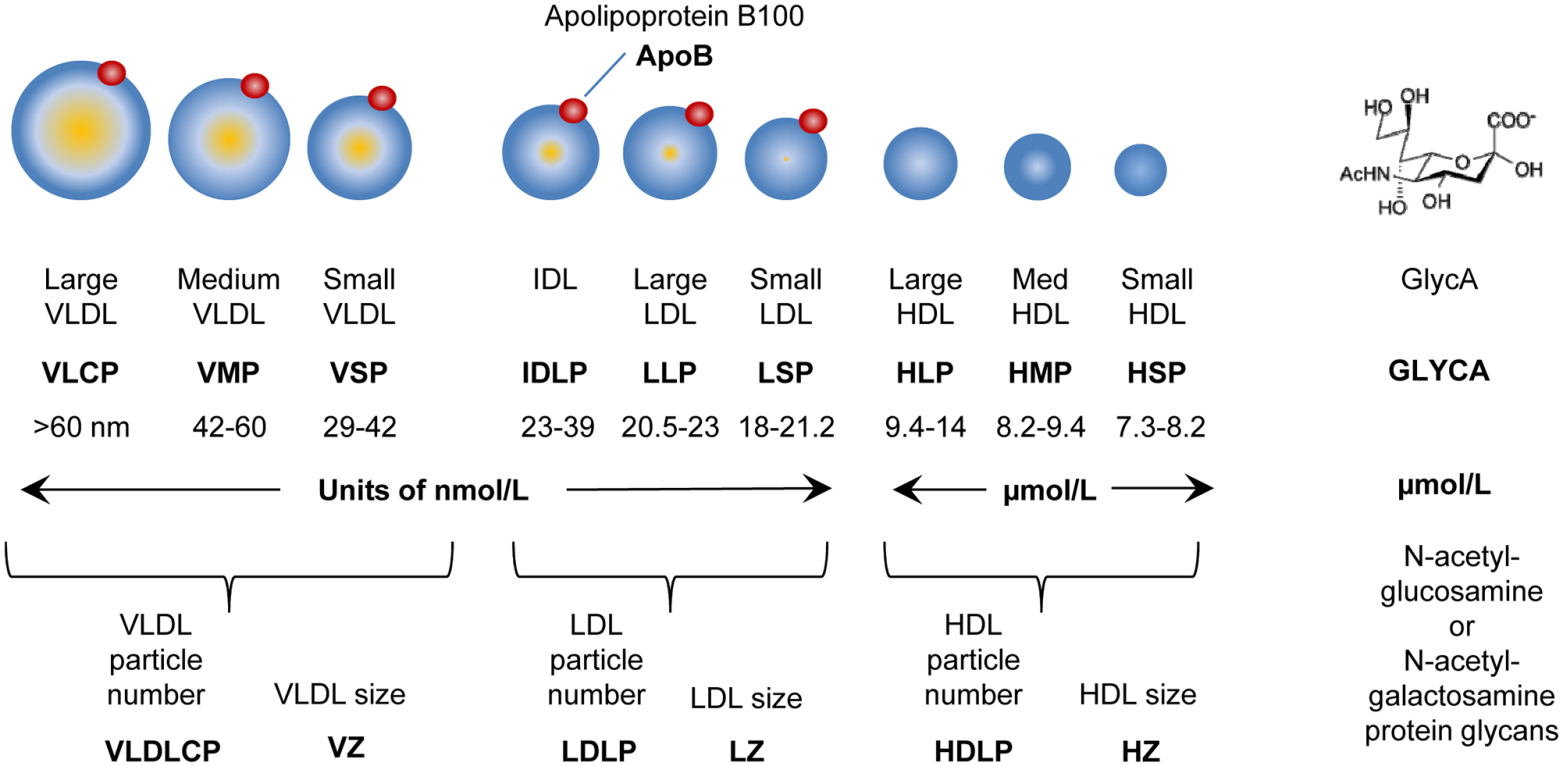
Schematic illustrating lipoproteins particles under investigation in this study.

Lipoprotein profiles consisting of VLDL, IDL, LDL and HDL concentration and particle size (Fig 1) from 70 class III obese females undergoing bariatric surgery and 9 lean female subjects were measured by H-NMR spectroscopy. Of our 70 class III obese females, 54 were Caucasian and 16 were African American. While the cohorts did not differ in demographics such as body weight, BMI, or insulin sensitivity, African American women exhibited a significant reduction in liver enzymes (Table 1). When lipid profiles were compared, African Americans exhibited a less atherogenic lipoprotein profiles including decreased levels of triglycerides, VLDL, and small LDL as well as improved LDL and HDL size ratios (Table 2).

**Table 1:** Baseline characteristics of class III obese females stratified by ethnicity.

	Caucasian	African Americans	*P*-value
**n**	54	16	
**Age**	43.4 ± 1.2	39.5 ± 2.2	0.116
**Weight (kg)**	117.3 ± 2.4	122.0 ± 6.0	0.711
**BMI (kg/m** ^**2**^ **)**	43.8 ± 0.8	43.9 ± 2.0	0.566
**Insulin (μU/mL)**	22.9 ± 1.7	19.9 ± 2.7	0.426
**Glucose (mg/dL)**	102 ± 2.7	98.2 ± 7.1	0.121
**HOMA2-IR (AU)**	2.9 ± 0.2	2.5 ± 0.3	0.365
**AST (normal range: 0–65 U/mL)**	29.3 ± 3.1	18.7 ± 1.4	0.074
**ALT (normal range: 0–65 U/mL)**	33.2 ± 3.0	15.8 ± 0.9	0.003*
**Alkaline Phosphatase (U/L)**	80.5 ± 3.0	82.4 ± 6.3	0.857
**Albumin (g/dL)**	4.1 ± 0.04	4.1 ± 0.1	0.624
**Platelets (1000/mm** ^**3**^ **)**	276.1 ± 7.9	282.2 ± 9.8	0.632
**Bilirubin (mg/dL)**	0.5 ± 0.03	0.4 ± 0.03	0.874

*Statistically significant changes; Data presented as mean ± SEM. AU, arbitrary units. BMI, body mass index; HOMA2-IR, updated homeostasis model assessment of insulin resistance; AST, aspartate aminotransferase; ALT, alanine aminotransferase; NAS, NAFLD Activity Score.

**Table 2:** Lipid and lipoprotein measures in class III obese females stratified by ethnicity.

	Caucasian	African American	*P*-value
*n*	54	16	
**NMR calculated lipids**			
Triglycerides (mg/dL)	157.7 ± 7.5	78.3 ± 4.7	< 0.001*
HDL cholesterol (mg/dL)	44.3 ± 1.2	51.9 ± 4.0	0.049*
VLDL & chylomicron TG (mg/dL)	123.5 ± 7.4	44.0 ± 3.9	< 0.001*
Triglycerides/HDL-C ratio	4.2 ± 0.2	1.7 ± 0.5	< 0.001*
**Lipoprotein particles**			
TOTAL VLDL (nmol/L)	71.4 ± 4.0	30.6 ± 3.3	< 0.001*
*Large VLDL & chylomicrons*	8.3 ± 0.7	2.0 ± 0.3	< 0.001*
*Medium VLDL*	26.7 ± 2.4	8.1 ± 1.3	< 0.001*
*Small VLDL*	36.3 ± 2.1	20.5 ± 2.9	< 0.001*
TOTAL LDL (nmol/L)	1,256 ± 57.7	1,083 ± 69.2	0.134
*IDL*	118.8 ± 12.3	85.1 ± 10.7	0.235
*Large LDL*	348 ± 35	566 ± 77	0.012*
*Small LDL*	789 ± 47	431 ± 77	0.001*
TOTAL HDL (μmol/L)	33.9 ± 0.8	33.3 ± 1.4	0.897
*Large HDL*	3.7 ± 0.2	6.6 ± 1.0	0.007*
*Medium HDL*	7.7 ± 0.7	9.1 ± 1.4	0.407
*Small HDL*	20.4 ± 0.8	16.4 ± 1.1	0.015
**ApoB100 (mg/dL)**	113.7 ± 3.7	96.1 ± 7.8	0.063
**Lipoprotein particle size** (nm)			
*VLDL*	53.9 ± 0.9	47.0 ± 1.8	0.001*
*LDL*	20.4 ± 0.1	20.8 ± 0.1	0.011*
*HDL*	8.9 ± 0.04	9.3 ± 0.1	0.005*
**Particle ratios**			
*LDL small/large ratio*	4.8 ± 0.8	2.6 ± 1.4	0.004*
*HDL small/large ratio*	6.5 ± 0.5	3.7 ± 0.7	0.001*
**Lipoprotein Insulin Resistance Score**	67.1 ± 2.6	37.6 ± 3.1	< 0.001*

*Statistically significant changes; Data presented as mean ± SEM. AU, arbitrary units.

Liver histology reports were available for 51 women (41 Caucasians and 10 African Americans). Class III obese females were stratified into subcohorts based on liver biopsy-determined NAFLD diagnoses of histologically normal (non-steatotic; n = 18), simple steatosis (SS; n = 16), or NASH (n = 17) via NAFLD Activity Score (NAS) and histological determination by pathologist as simple steatosis or steatohepatitis. Weight, BMI, and fasting glucose levels were similar across the three obese groups. NASH females were slightly older and exhibited increased levels of AST, ALT and fasting insulin, and insulin resistance per HOMA2-IR, but these results were adjusted for age (Table 3). Histology was not available for lean women, but demographics and lipoprotein profiles were compared to obese individuals with normal histology.

**Table 3:** Baseline characteristics of class III obese females stratified by histology.

	Leans	Obese Normal	Obese SS	Obese NASH	*P*-Value
**n**	9	18	16	17	-
**Gender (F/M)**	9/0	18/0	16/0	17/0	-
**Caucasian/African American**	7/2	13/5	5-Nov	17/0	-
**Age**	40.2 ± 2.0	38.8 ± 1.4	43.7 ± 2.9	47.2 ± 6.3^b^	0.009
**Weight (kg)**	59.5 ± 2.2****	122.7 ± 5.2	116.0 ± 4.7	116.8 ± 2.9	0.553
**BMI (kg/m** ^**2**^ **)**	22.5 ± 0.8****	45.4 ± 1.3	43.1 ± 1.3	43.9 ± 1.6	0.400
**Fasting insulin (μU/mL)**	7.6 ± 1.0****	18.7 ± 1.8	20.3 ± 3.2	28.8 ± 3.5^a^	0.026
**Fasting glucose (mg/dL)**	81.6 ± 1.9***	96.4 ± 2.6	102.9 ± 6.5	109.8 ± 6.2	0.241
**HOMA2-IR (AU)**	0.9 ± 0.2***	2.4 ± 0.2	2.5 ± 0.4	3.7 ± 0.4^c^	0.018
**AST (normal range: 0–65 U/mL)**	-	20.2 ± 1.5	26.5 ± 3.5	46.6 ± 9.0^a^	0.022
**ALT (normal range: 0–65 U/mL)**	-	22.3 ± 2.7	29.3 ± 4.9	49.2 ± 7.1^b^	0.009
**Alkaline Phosphatase (U/L)**	-	72.6 ± 5.0	91.1 ± 7.0	82.3 ± 4.3	0.271
**Albumin (g/dL)**	-	4.1 ± 0.1	4.2 ± 0.1	4.1 ± 0.1	0.936
**Bilirubin (mg/dL)**	-	0.5 ± 0.1	0.5 ± 0.1	0.5 ± 0.1	0.532
**NAS**	-	0.8 ± 0.2	2.7 ± 0.3^a^	5.0 ± 0.2^d^	<0.001

Data presented as mean ± SEM. AU, arbitrary units. Asterisks represent significant difference between lean and obese:

***P<0.001

****P<0.0001

One-way ANOVA P values among obese groups (far-right). Dunn’s Multiple Comparison test results for obese group:

^a^P <0.05 vs. normal

^b^P <0.01 vs. normal

^c^P <0.05 vs. SS

^d^P <0.001 vs. normal

Abbreviations: BMI, body mass index; HOMA2-IR, updated homeostasis model assessment of insulin resistance; AST, aspartate aminotransferase; ALT, alanine aminotransferase; NAS, NAFLD Activity Score.

### Lipoprotein Profiles in Class III Obese Women by Histology

Compared to lean females, obese females with normal histology had an elevated HOMA2-IR, driven by increased fasting glucose and insulin levels (Table 3). Obese women with normal liver histology were more insulin resistant and had a more atherogenic lipid profile than lean females manifest by increased large and medium VLDL, increased total and small LDL, and increased small to large HDL ratio (Table 4). The Lipoprotein Insulin Resistance Score (LP-IR) was also elevated in the pathologically normal women compared to leans.

**Table 4:** Lipid and lipoprotein measures in class III obese females stratified by histology.

Variable	Lean	Obese Normal	Obese SS	Obese NASH	*P*-value
*n*	9	18	16	17	
**NMR calculated lipids**					
Triglycerides (mg/dL)	76.9 ± 2.9***	119.0 ± 12.9	157.0 ± 18.8	168.2 ± 9.9^a^	0.017
HDL cholesterol (mg/dL)	60.6 ± 4.2*	45.3 ± 2.6	51.0 ± 4.2	44.8 ± 1.8	0.58
VLDL & chylomicron TG (mg/dL)	42.9 ± 4.1***	85.1 ± 13.1	115.7 ± 17.4	136.8 ± 10.2^a^	0.016
Triglycerides/HDL-C ratio	1.3 ± 0.1****	2.8 ± 0.4	3.5 ± 0.6	3.9 ± 0.3	0.081
**Lipoprotein particles**					
TOTAL VLDL (nmol/L)	34.9 ± 4.0**	52.7 ± 8.5	63.5 ± 8.7	72.5 ± 5.6	0.165
*Large VLDL & chylomicrons*	1.0 ± 0.2***	4.7 ± 0.9	8.2 ± 1.4	10.4 ± 1.0^b^	0.002
*Medium VLDL*	7.4 ± 1.8**	11.2 ± 2.5	23.2 ± 4.8	25.3 ± 2.8	0.254
*Small VLDL*	26.5 ± 3.2	22.4 ± 2.6	32.1 ± 4.6	36.7 ± 3.2	0.109
TOTAL LDL (nmol/L)	853 ± 117**	1,177 ± 76	1,487 ± 101^a^	1,157 ± 141	0.05
*IDL*	104 ± 18	111 ± 17	140. ± 29	102 ± 15	0.839
*Large LDL*	675 ± 114*	457 ± 69	527 ± 73	264 ± 62^c^	0.018
*Small LDL*	72 ± 12****	608 ± 90	820 ± 115	789 ± 75	0.379
TOTAL HDL (μmol/L)	35.3 ± 1.7	31.7 ± 1.1	35.7 ± 1.9	35.2 ± 1.4	0.168
*Large HDL*	9.2 ± 0.9****	5.0 ± 0.7	5.2 ± 1.0	3.6 ± 0.4	0.452
*Medium HDL*	17.1 ± 1.6***	7.2 ± 1.1	6.9 ± 1.0	7.2 ± 1.1	0.979
*Small HDL*	9.0 ± 1.6****	17.5 ± 1.3	20.9 ± 1.6	21.6 ± 1.3	0.066
**ApoB100 (mg/dL)**	**-**	106.2 ± 5.1	120.8 ± 7.5	103.6 ± 6.1	0.127
**Lipoprotein particle size** (nm)					
*VLDL*	43.5 ± 0.7***	50.4 ± 1.1	54.0 ± 2.2	57.3 ± 1.1^b^	0.003
*LDL*	21.3 ± 0.1***	20.6 ± 0.1	20.6 ± 0.2	20.2 ± 0.1	0.053
*HDL*	9.8 ± 0.1****	9.1 ± 0.1	8.9 ± 0.1	8.9 ± 0.1	0.668
**Particle ratios**					
*LDL small/large ratio*	0.1 ± 0.0****	3.9 ± 1.5	3.9 ± 1.9	5.6 ± 1.1	0.047
*HDL small/large ratio*	1.1 ± 0.3****	4.9 ± 0.8	6.0 ± 1.0	6.9 ± 0.9	0.286
**Lipoprotein Insulin Resistance Score**	13.4 ± 1.7****	51.8 ± 5.3	63.2 ± 6.1	73.4 ± 2.8^a^	0.021

Data presented as mean ± SEM. AU, arbitrary units. Asterisks represent significant difference between lean and obese.

* P<0.05

**P<0.01

***P<0.001

****P<0.0001

One-way ANOVA P values among obese groups (far-right). Dunn’s Multiple Comparison test results for obese group:

^a^P < 0.05 vs. normal

^b^P < 0.01 vs. normal

^c^P < 0.05 vs. SS

Among obese women in the cohort with NAFLD, NASH patients exhibited higher HOMA2-IR driven primarily by a significant increase in fasting insulin compared to SS and normal females. Further, NMR determined lipoprotein profiles differed across histology. NASH females had significantly elevated levels of triglycerides, VLDL cholesterol, large VLDL particle number, and VLDL size compared to normal and SS females (Table 4). Women with SS had increased large LDL levels relative to normal females, while NASH patients interestingly had decreased large LDL levels. LP-IR was elevated in obese NASH relative to the obese with normal histology. There were no differences in HDL particle number, LDL size, HDL size, or ApoB100 concentration in the obese cohort as a whole. We also examined the potential association between lipoprotein measures and NAS using linear regression with NAS score as outcome and lipoprotein measure as predictor. Other adjusted clinical variables include age, race, weight and BMI. VLDL size, large VLD concentration as well as serum AST, LP-IR, ALT, TG were found to be positively associated with NAS score. LDL size was found to be inversely associated with NAS score. Furthermore, we also examined the association between each individual component of NAS and lipoprotein measures. The full results of this analysis can be viewed in data in Table B in S1 File.

### Impact of Cohort Selection on Lipoprotein Profiles in NAFLD

When the 41 female Caucasians with NAFLD were examined without the African Americans in the cohort, additional differences in lipoprotein profiles across pathology emerged compared to those seen in the cohort as a whole. In the Caucasian cohort, 17 had NASH, 11 had SS, and 13 had normal liver histology (Table 5). Caucasian women with SS showed significant increases in TG concentration, VLDL concentration, and VLDL size versus normal histology (Table 4). However, women with NASH demonstrated a decline in TG and VLDL concentrations relative to SS females, and this was accompanied by significant reductions in total LDL and apoB100 particle numbers (Table 4). Simultaneously, there were increases in HOMA2-IR and insulin in NASH versus SS, and elevated LP-IR in NASH versus normal.

**Table 5:** Lipid and lipoprotein measures in class III obese Caucasian females stratified by histology.

Variable	Obese Normal	Obese SS	Obese NASH	*P*-value
*n*	13	11	17	
**HOMA2-IR**	2.5 ± 0.3	2.0 ± 0.5	3.6 ± 0.5^d^	0.004
Insulin	18.6 ± 1.9	17.2 ± 4.1	28.8 ± 3.8^d^	0.006
Glucose	95.1 ± 3.0	98.0 ± 4.5	109.8 ± 6.2	0.099
**NMR calculated lipids**				
Triglycerides (mg/dL)	136.5 ± 15.8	195.5 ± 19.2^a^	168.2 ± 9.9	0.034
HDL cholesterol (mg/dL)	42.8 ± 2.6	46.4 ± 3.7	44.8 ± 1.8	0.427
VLDL & chylomicron TG (mg/dL)	102.7 ± 16.5	152.7 ± 17.2	136.8 ± 10.2	0.0651
**Lipoprotein particles**				
TOTAL VLDL (nmol/L)	65.3 ± 10.3	81.6 ± 8.7	72.48 ± 5.6	0.895
*Large VLDL & chylomicrons*	5.8 ± 1.2	11.2 ± 1.4^b^	10.4 ± 1.0^b^	0.006
*Medium VLDL*	28.0 ± 6.8	31.1 ± 6.0	25.3 ± 2.8	0.736
*Small VLDL*	31.5 ± 4.4	39.2 ± 5.7	36.7 ± 3.2	0.472
TOTAL LDL (nmol/L)	1241 ± 84	1636 ± 117	1157 ± 101^c^	0.014
*IDL*	114 ± 22	173 ± 41	102 ± 15	0.377
*Large LDL*	417 ± 68	443 ± 85	264 ± 62	0.076
*Small LDL*	708 ± 98	1019 ± 119	789 ± 75	0.228
TOTAL HDL (μmol/L)	31.6 ± 1.5	35.5 ± 2.6	35.3 ± 1.4	0.264
*Large HDL*	4.0 ± 0.6	3.6 ± 0.6	3.6 ± 0.4	0.815
*Medium HDL*	6.4 ± 1.1	6.1 ± 1.1	7.2 ± 1.1	0.899
*Small HDL*	19.3 ± 1.2	22.7 ± 2.2	21.6 ± 1.4	0.311
**ApoB100 (mg/dL)**	111.5 ± 4.7	131.0 ± 7.6	103.6 ± 6.1^c^	0.020
**Lipoprotein particle size** (nm)				
*VLDL*	50.6 ± 1.4	57.0 ± 1.9^b^	57.3 ± 1.0^b^	0.002
*LDL*	20.5 ± 0.2	20.4 ± 0.2	20.2 ± 0.1	0.182
*HDL*	8.9 ± 0.1	8.7 ± 0.08	8.9 ± 0.09	0.497
**Particle ratios**				
*LDL small/large ratio*	3.7 ± 1.2	5.9 ± 3.0	5.6 ± 1.1	0.511
*HDL small/large ratio*	5.7 ± 0.9	6.6 ± 0.6	7.2 ± 0.9	0.459
**Lipoprotein Insulin Resistance Score**	45.9 ± 6.4	56.6 ± 7.2	73.4 ± 4.7^a^	0.027

Data presented as mean ± SEM. AU, arbitrary units. P values represent difference between groups.

^a^P < 0.05 vs. normal

^b^P < 0.01 vs. normal

^c^P < 0.05 vs. SS

^d^P < 0.01 vs. SS

Interestingly, despite enrolling every African American woman who came through clinic during our proposed study period, none of the females had a diagnosis of NASH. In this small African American cohort, 5 had SS and 5 had non-steatotic normal liver histology. There were no differences in demographics or lipoprotein profiles (Table 6) between the groups.

**Table 6:** Lipid and lipoprotein measures in class III obese African American females stratified by histology.

Variable	Obese Normal	Obese SS	*P*-value
*n*	5	5	
**HOMA2-IR**	2.2 ± 0.5	3.4 ± 0.6	0.222
Insulin	18.7 ± 4.8	26.6 ± 5.2	0.530
Glucose	99.8 ± 5.2	113.6 ± 18.9	0.917
**NMR calculated lipids**			
Triglycerides (mg/dL)	77.0 ± 11.5	80.0 ± 7.7	0.401
HDL cholesterol (mg/dL)	51.2 ± 6.5	60.2 ± 10.2	0.802
VLDL & chylomicron TG (mg/dL)	42.96 ± 8.6	41.72 ± 5.1	0.667
**Lipoprotein particles**			
TOTAL VLDL (nmol/L)	22.66 ± 5.4	27.5 ± 3.5	0.421
*Large VLDL & chylomicrons*	2.3 ± 0.8	2.3 ± 0.7	0.917
*Medium VLDL*	8.5 ± 2.0	7.2 ± 2.0	0.841
*Small VLDL*	11.8 ± 4.1	18.0 ± 4.4	0.421
TOTAL LDL (nmol/L)	1026 ± 168	1191 ± 139	0.691
*IDL*	103 ± 27	74 ± 16	0.548
*Large LDL*	555 ± 184	694 ± 131	0.691
*Small LDL*	367 ± 180	422 ± 155	0.841
TOTAL HDL (μmol/L)	31.8 ± 1.6	36.1 ± 3.0	0.295
*Large HDL*	7.4 ± 1.7	8.5 ± 2.3	0.737
*Medium HDL*	9.3 ± 2.6	8.5 ± 2.3	0.691
*Small HDL*	13.04 ± 2.5	17.4 ± 1.4	0.310
**ApoB100 (mg/dL)**	92.6 ± 12.5	98.4 ± 13.4	0.472
**Lipoprotein particle size** (nm)			
*VLDL*	49.5 ± 2.7	48.0 ± 4.8	0.714
*LDL*	20.6 ± 0.3	21.1 ± 0.2	0.139
*HDL*	9.5 ± 0.2	9.4 ± 0.2	0.325
**Particle ratios**			
*LDL small/large ratio*	5.3 ± 4.6	0.9 ± 0.4	0.366
*HDL small/large ratio*	2.9 ± 1.4	2.7 ± 0.6	0.916
**Lipoprotein Insulin Resistance Score**	33.8	36.2	0.397

Data presented as mean ± SEM. AU, arbitrary units.

### Comparing African Americans and Caucasians with SS

**Fig 2:**
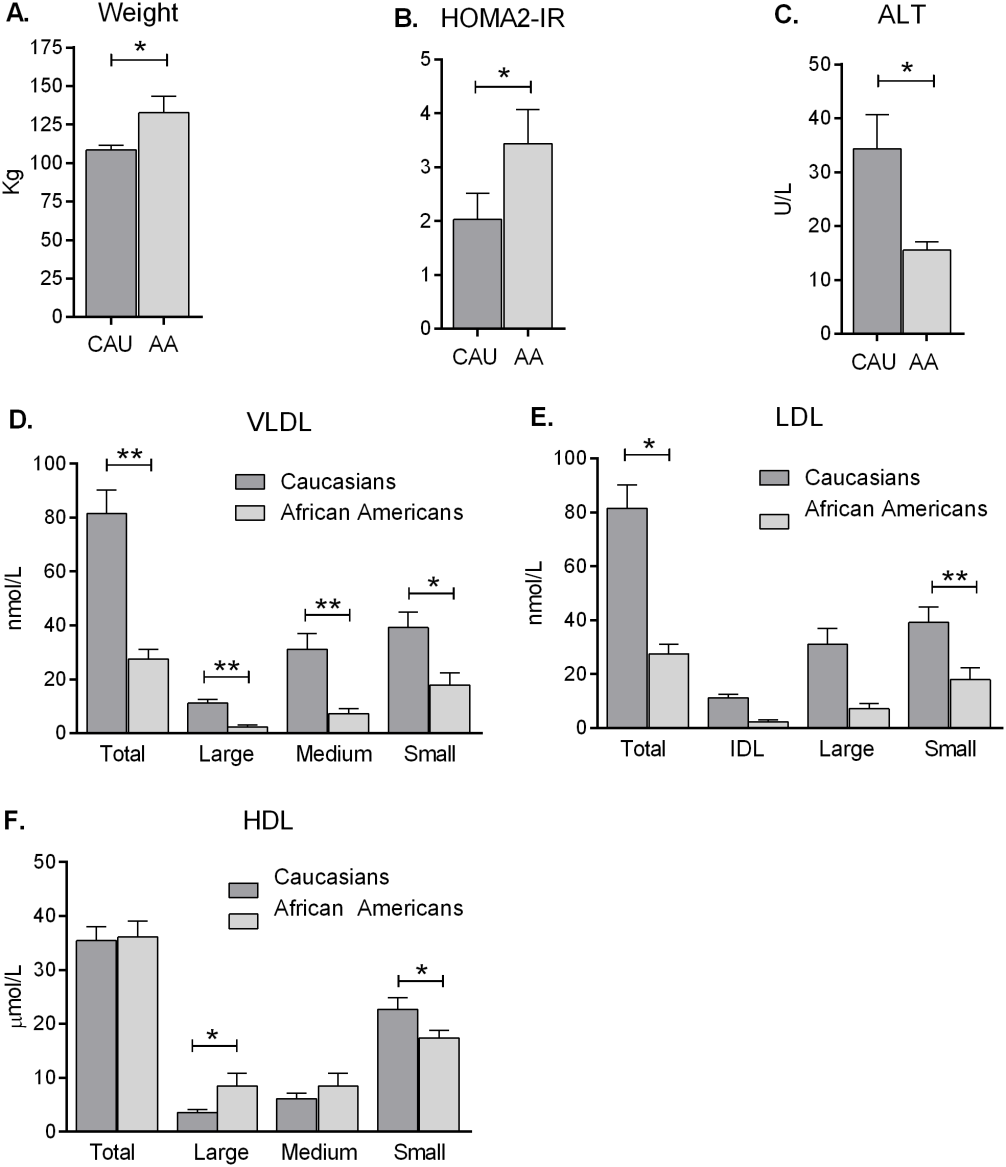
Ethnic Lipoparticle Comparisons. African American women with simple steatosis had less atherogenic lipoprotein profiles relative than Caucasian women with simple steatosis. **A)** African Americans with simple steatosis (SS) were heavier (*P<0.05) and more insulin resistant **B**) as measured by homeostatic model assessment index 2 (HOMA2-IR, *P<0.05), yet displayed lower triglycerides (***P<0.001, not shown) and **C**) lower alanine aminotransferase (ALT, *P<0.05) than Caucasian SS. **D**) African Americans with SS displayed lower total very-low-density lipoprotein (VLDL) and VLDL sub-fractions (**P<0.01 for all, except small VLDL particle concentration, *P<0.05), **E**) decreased total and small low-density lipoprotein (LDL; *P<0.05 and **P<0.01, respectively), and **F**) improved high-density lipoprotein (HDL) profiles (increased large HDL, p = 0.002, and lower small LDL, P = 0.04) relative to Caucasian SS (E-F). Values are mean ± SEM. Data were analyzed by one-way ANOVA, followed by Mann-Whitney t Tests.

Despite having higher BMI’s and insulin resistance measured by HOMA2-IR compared to Caucasians with SS, the African American women with SS had decreased triglycerides and liver enzymes (Fig 2A–2C). African Americans with SS had lower total VLDL and all sub-fractions (Fig 2D) and decreased total and small LDL compared to Caucasians (Fig 2E). African Americans also had improved HDL profiles as evidenced by increased large HDL and less small HDL particle numbers compared to Caucasians with SS (Fig 2F). Further, Caucasians with SS had a much higher LP-IR score than African Americans with SS (Table 6).

## Discussion

This study in a controlled population of histologically proven NAFLD identified novel changes in fasting lipoprotein subclass particle number and size that delineated simple steatosis from NASH. Namely, we found that circulating VLDL particle number, VLDL size, and TG content were increased in SS relative to weight-matched obese normal females, whereas NASH females trended toward decreased VLDL with significant declines in LDL particle number and apoB100 concentrations compared to SS. We also identified disparities in the relationship between lipoprotein profiles and NAFLD in African American women relative to Caucasians. The relationship parallels the differential prevalence of dyslipidemia across ethnicities and may explain the variation in studies of NAFLD epidemiology and dyslipidemia in the literature to date [9]. These findings suggest a role for monitoring lipoprotein profiles in NAFLD progression, but that ethnicity should be considered in the process [33].

Individual lipoprotein parameters such as increased small LDL, small HDL, and ApoB100 have been associated with increased atherogenic risk and diabetes independent of obesity, but less is known about lipoprotein characteristics relative to NAFLD progression [28, 29, 34–36]. Given the liver is the site of a majority lipoprotein secretion and uptake, namely VLDL and LDL, alterations in lipoprotein profiles may reflect changes in NAFLD progression [1, 37–40]. Studies using imaging such as CT or MRI [10] to diagnose NAFLD found that the lipoprotein profile changes accompanying NAFLD are similar to the dyslipidemia that precedes the onset of T2D including increased triglycerides, large VLDL, VLDL size, and small LDL, and decreased large HDL [5, 12–14, 18]. Further, they found that triglycerides and total cholesterol increase and HDL decreases with hepatic fat. However, these studies were insufficient to describe NASH, as only liver biopsy can detect the inflammation, fibrosis, and ballooning that distinguish NASH from steatosis [9, 32, 41, 42]. Other studies have evaluated lipoprotein using AST and ALT as surrogate markers of hepatic dysfunction, but these enzymes are normal in up to 78% of patients with NAFLD and thus cannot be used to accurately diagnose the disease or distinguish steatosis from NASH [20–22].

Of the studies in biopsy confirmed NASH, plasma lipoparticle analyses by HPLC or ultracentrifugation have shown an increase in total triglycerides, VLDL, LDL, and ApoB100 and a decrease in HDL in NASH relative to normal livers [4]. Studies of steatosis versus NASH however have been less congruent, as some show decreases in VLDL (and HDL), and others an increase. Recently, the use of NMR-derived lipoprotein profiles revealed previously undetected changes in particle number and size that are associated with metabolic disease [34]. We are only the second study to employ this technology to compare biopsy driven NAFLD diagnoses to lipoprotein profiles [43]., but this other work did not use the standard subclass classifications employed by the existing literature and available commercial methods [25], thus limiting its applicability [24]. In addition, the group did not report differences between SS and NASH, only differences relative to normal histology, nor did they stratify their cohort by ethnicity or gender. Similar to ethnicity, the relationship between dyslipidemia and metabolic diseases such as diabetes, cardiovascular disease, and NAFLD differs by gender, with females displaying improved lipid profiles relative to men [11, 44]. Hence, the study may have missed significant differences in lipoprotein subclass profiles across pathology due to cohort heterogeneity.

In this study we found that changes in steatosis versus NASH were most pronounced when Caucasian women were examined alone. Among Caucasians, increased VLDL size and TG content were the first changes associated with the development of NAFLD, similar to other in ethnically heterogeneous reports [15, 16, 45]. Here progression from SS to NASH was accompanied by more global dysfunction, with decreased total LDL and apoB100 production and increased insulin resistance. These findings suggest a role for deranged lipid production in disease progression, in particular ApoB100 [15, 16, 46]. ApoB100 is a lipid-binding protein produced in the liver that is necessary for the assembly and secretion of VLDL [39]. Because one apoB100 is required for each VLDL particle, its availability determines hepatic lipoprotein production, thus deficiency in the protein synthesis or release could contribute to hepatic TG retention [5]. Fujita et al. came to a similar conclusion when they found that VLDL and ApoB100 mRNA was decreased in NASH relative to simple steatosis patients, but they found that LDL was increased. However, they did not evaluate lipoprotein parameters via H-NMR nor did they stratify their results by gender, both of which might explain the different results [16].

In addition to the decline in lipoprotein parameters in NASH, we found differences in demographics and lipid profiles between African Americans and Caucasian women with SS. Here none of the African American women had NASH. This is similar to the epidemiological literature which reports decreased rates of NAFLD and NASH among African Americans, an observation that has been attributed to variation in genetics and body fat distribution [47]. We found that African Americans with SS weighed more and were more insulin resistant than Caucasians with SS, yet had a less atherogenic lipid profile. This reflects the “triglyceride paradox” of improved dyslipidemia in African Americans relative to other ethnicities despite higher rates of obesity and insulin resistance [48]. Attempts to explain this discrepancy have implicated increased lipoprotein lipase (LPL) activity and lower levels of the LPL inhibitory protein, apolipoprotein CII, in African Americans as a cause [47, 49] as well as an apoB100 gene variant that is associated with VLDL diameter in Caucasians and Hispanics, but not African Americans [50]. While the mechanisms are yet to be elucidated, these findings point to potential ethnic differences in hepatic lipid metabolism [44, 47, 50].

A final novel facet of this study was the use of the lipoprotein-insulin resistance (LP-IR) score to quantify the relationship between dyslipidemia and insulin sensitivity. The importance of lipids in the pathogenesis of insulin resistance has been highlighted by Randle et al. who described a glucose-fatty cycle where increased byproducts of lipid oxidation lead to inhibition of glucose uptake in type I diabetics [26]. Other studies utilizing H-NMR techniques found significant associations between lipoprotein profile constituents and insulin sensitivity [28, 29]. Six parameters in particular have been used to create an overall picture of insulin resistance known as the LP-IR Score [26, 30]. Here we found a positive relationship between LP-IR and HOMA2-IR including a significant increase in NASH females. Further, increases in LP-IR in Caucasians accompanied or preceded increases in HOMA2-IR and were associated with progression to NASH. Given dyslipidemia’s tendency to precede the onset of frank diabetes, the LP-IR score and the lipocentric view of insulin resistance might be of use in the early identification of T2D or NASH [5], but additional studies are needed to further validate this new technique.

This study has some limitations. While liver biopsy remains the “gold standard” for assessing NAFLD severity, the procedure carries risk and therefore was not available for all females [19]. This also rendered our study cohort smaller than those used in larger population-driven studies. Our African American population is especially small, but is reflective of the larger bariatric surgery population, which is currently predominately Caucasian [51, 52]. This work should be repeated in a larger, ethnically diverse cohort of both sexes. Similar to ethnicity, differences in NAFLD severity and dyslipidemia are described in males versus females, thus our decision to recruit only females [53]. Other considerations include more detailed analysis of insulin resistance via hyperinsulinemic euglycemic clamp or lipoprotein production via tracer studies. It would also be useful to assess liver histology longitudinally, but unfortunately, non-clinically indicated liver biopsies are rarely warranted based on ethical concerns.

In conclusion, this study identified differences in total lipoprotein and subclass levels across NAFLD severity that differed in Caucasians versus African American women. The combination of NMR lipoprotein analysis and biopsy driven NASH diagnoses provided the opportunity to appreciate previously undetected changes in lipoprotein profiles, namely changes in VLDL, LDL, and apoB100 that can be used to delineate steatosis from NASH in Caucasian women. These findings suggest a potential role for lipoprotein subclass concentration and size as markers of NAFLD progression, particularly apoB100. Monitoring for relative reductions in these parameters, as well as changes in the LP-IR index, may enable clinicians to better identify and monitor those at increased risk for developing NASH. However, if this strategy is to be employed clinically, our observations in African Americans with SS highlight the need for separate parameters for each ethnicity, as the relationship between dyslipidemia and NAFLD does not appear to be racially homogenous.

## Supporting Information

S1 File (DOCX)
