# Supplementary material for: Lipoprotein Profiles in Class III Obese Caucasian and African American Women with Nonalcoholic Fatty Liver Disease
Source: PLoS One. 2015 Nov 23;10(11):e0142676. doi: 10.1371/journal.pone.0142676 (PMC4657895; doi:10.1371/journal.pone.0142676)
Supplement: S1 File — (DOCX) [file pone.0142676.s001.docx]

**Table A: NAFLD Activity Score (NAS)**

| ID | Ethnicity | Age | Inflammation | Steatosis | Ballooning | NAS | Fibrosis | Category |
| --- | --- | --- | --- | --- | --- | --- | --- | --- |
| R54 | CAU | 37 | 0 | 1 | 0 | 1 | 0 | Normal |
| R55 | CAU | 40 | 0 | 0 | 0 | 0 | 0 | Normal |
| R58 | CAU | 54 | 1 | 1 | 0 | 2 | 0 | SS |
| R60 | CAU | 54 | 1 | 2 | 1 | 4 | 3 | NASH |
| 63 | CAU | 50 | 1 | 3 | 1 | 5 | 1 | NASH |
| AG5 | CAU | 32 | 0 | 1 | 0 | 1 | 0 | Normal |
| AG6 | CAU | 41 | 1 | 0 | 0 | 1 | 0 | Normal |
| AG7 | CAU | 48 | 1 | 2 | 2 | 5 | 3 | NASH |
| AG8 | CAU | 48 | 1 | 3 | 2 | 6 | 3 | NASH |
| AG10 | CAU | 42 | 1 | 1 | 1 | 3 | 0 | SS |
| AG13 | CAU | 29 | 1 | 3 | 1 | 5 | 1 | NASH |
| AG14 | CAU | 41 | 0 | 0 | 0 | 0 | 0 | Normal |
| AG16 | CAU | 36 | 0 | 0 | 0 | 0 | 0 | Normal |
| AG17 | CAU | 46 | 1 | 3 | 1 | 5 | 0 | NASH |
| AG18 | CAU | 23 | 1 | 2 | 2 | 5 | 0 | NASH |
| AG19 | CAU | 48 | 0 | 1 | 0 | 1 | 0 | Normal |
| AG22 | CAU | 60 | 1 | 2 | 1 | 4 | 1 | NASH |
| R70 | CAU | 49 | 1 | 3 | 2 | 6 | 2 | NASH |
| G3 | CAU | 54 | 1 | 3 | 2 | 6 | 0 | NASH |
| R87 | AA | 39 | 0 | 1 | 0 | 1 | 0 | Normal |
| G6 | CAU | 49 | 0 | 0 | 1 | 1 | 0 | Normal |
| R102 | CAU | 40 | 0 | 1 | 0 | 1 | 0 | Normal |
| 105 | AA | 58 | 0 | 1 | 1 | 2 | 0 | SS |
| 108 | AA | 28 | 0 | 0 | 0 | 0 | 0 | Normal |
| 109 | CAU | 57 | 1 | 2 | 1 | 4 | 0 | NASH |
| 111 | CAU | 51 | 1 | 2 | 2 | 5 | 2 | NASH |
| 114 | AA | 33 | 0 | 1 | 0 | 1 | 0 | Normal |
| 116 | AA | 44 | 0 | 1 | 0 | 1 | 0 | Normal |
| G8 | CAU | 51 | 0 | 0 | 0 | 0 | 0 | Normal |
| G9 | CAU | 28 | 1 | 1 | 1 | 3 | 0 | SS |
| 84 | CAU | 47 | 1 | 2 | 1 | 4 | 0 | NASH |
| Abbreviations: CAU – Caucasian; AA – African American | | | | | | | | |

Subject IDs, ethnicities (CAU – Caucasian; AA – African American), ages and individual scores (inflammation, steatosis, ballooning) used to derive the NAFLD Activity Score (NAS). Fibrosis scores and categorical membership of normal, simple steatosis (SS) or nonalcoholic steatohepatitis (NASH).

| Table B. Regression analysis of NAS components and study variables | | | | | | | | | | | | | | | |
| --- | --- | --- | --- | --- | --- | --- | --- | --- | --- | --- | --- | --- | --- | --- | --- |
|  | **Inflammation** | | | **Fat** | | | **Ballooning** | | | **Score** | | | **Fibrosis** | | |
| Variable | **Beta** | **S.E.** | ***p*** | **Beta** | **S.E.** | ***p*** | **Beta** | **S.E.** | ***p*** | **Beta** | **S.E.** | **pvalue** | **Beta** | **S.E.** | ***p*** |
| Albumin | 0.193 | 0.396 | 0.634 | 0.705 | 0.608 | 0.265 | 0.835 | 0.551 | 0.152 | 1.733 | 1.334 | 0.215 | 0.523 | 0.619 | 0.412 |
| AlkPhos | 0.002 | 0.005 | 0.744 | -0.006 | 0.008 | 0.480 | -0.006 | 0.008 | 0.474 | -0.010 | 0.019 | 0.600 | -0.013 | 0.008 | 0.126 |
| ALT | 0.014 | 0.008 | 0.104 | 0.018 | 0.013 | 0.203 | 0.028 | 0.012 | **0.039** | 0.060 | 0.028 | 0.055 | 0.026 | 0.010 | **0.024** |
| ApoB100 | 0.002 | 0.004 | 0.713 | -0.006 | 0.009 | 0.522 | 0.007 | 0.006 | 0.265 | 0.003 | 0.019 | 0.882 | -0.001 | 0.008 | 0.898 |
| AST | 0.013 | 0.009 | 0.156 | 0.016 | 0.014 | 0.266 | 0.034 | 0.012 | **0.014** | 0.063 | 0.030 | 0.052 | 0.032 | 0.010 | **0.005** |
| Bilirubin | -0.728 | 0.609 | 0.253 | -0.883 | 0.985 | 0.386 | -0.622 | 0.947 | 0.522 | -2.234 | 2.177 | 0.323 | 1.779 | 0.894 | 0.068 |
| Glucose | 0.009 | 0.007 | 0.227 | 0.021 | 0.015 | 0.186 | 0.014 | 0.011 | 0.218 | 0.044 | 0.030 | 0.161 | 0.013 | 0.014 | 0.381 |
| GlycA | 0.001 | 0.002 | 0.549 | -0.003 | 0.003 | 0.342 | -0.001 | 0.003 | 0.673 | -0.004 | 0.007 | 0.608 | -0.002 | 0.003 | 0.467 |
| HDLP | -0.011 | 0.014 | 0.458 | 0.023 | 0.033 | 0.495 | -0.019 | 0.024 | 0.422 | -0.007 | 0.065 | 0.909 | -0.025 | 0.030 | 0.419 |
| HDL/SL | 0.064 | 0.034 | 0.073 | 0.140 | 0.077 | 0.083 | 0.061 | 0.060 | 0.324 | 0.265 | 0.152 | 0.096 | 0.115 | 0.072 | 0.126 |
| HLP | -0.091 | 0.058 | 0.128 | -0.017 | 0.139 | 0.902 | -0.169 | 0.094 | 0.086 | -0.277 | 0.263 | 0.303 | -0.046 | 0.125 | 0.717 |
| HMP | -0.020 | 0.021 | 0.345 | -0.030 | 0.048 | 0.542 | -0.008 | 0.035 | 0.815 | -0.058 | 0.093 | 0.539 | -0.029 | 0.044 | 0.509 |
| HOMA | 0.062 | 0.072 | 0.394 | 0.222 | 0.153 | 0.159 | 0.206 | 0.109 | 0.071 | 0.491 | 0.301 | 0.117 | 0.312 | 0.111 | **0.010** |
| HSP | -0.001 | 0.018 | 0.975 | 0.033 | 0.041 | 0.431 | -0.009 | 0.030 | 0.763 | 0.023 | 0.080 | 0.776 | 0.000 | 0.038 | 0.990 |
| HZ | -0.343 | 0.292 | 0.252 | -0.250 | 0.686 | 0.719 | -0.537 | 0.485 | 0.279 | -1.130 | 1.311 | 0.398 | 0.319 | 0.619 | 0.612 |
| IDLP | 0.000 | 0.002 | 0.990 | 0.003 | 0.004 | 0.480 | 0.002 | 0.003 | 0.392 | 0.005 | 0.008 | 0.496 | 0.005 | 0.003 | 0.127 |
| Insulin | 0.013 | 0.008 | 0.128 | 0.029 | 0.018 | 0.111 | 0.030 | 0.012 | **0.015** | 0.072 | 0.033 | **0.037** | 0.036 | 0.012 | **0.009** |
| LDLP | 0.000 | 0.000 | 0.154 | 0.000 | 0.001 | 0.879 | 0.001 | 0.000 | 0.067 | 0.001 | 0.001 | 0.286 | 0.000 | 0.000 | 0.460 |
| LDL/SL | 0.056 | 0.026 | **0.046** | 0.084 | 0.064 | 0.202 | 0.109 | 0.042 | **0.017** | 0.248 | 0.117 | **0.045** | 0.038 | 0.059 | 0.525 |
| LLP | 0.000 | 0.000 | 0.530 | -0.001 | 0.001 | 0.118 | 0.000 | 0.001 | 0.578 | -0.002 | 0.002 | 0.250 | 0.000 | 0.001 | 0.586 |
| LPIR | 0.013 | 0.004 | **0.008** | 0.020 | 0.011 | 0.086 | 0.017 | 0.008 | **0.033** | 0.050 | 0.020 | **0.021** | 0.007 | 0.010 | 0.513 |
| LSP | 0.001 | 0.000 | **0.007** | 0.001 | 0.001 | 0.148 | 0.002 | 0.000 | **0.002** | 0.003 | 0.001 | **0.012** | 0.001 | 0.001 | 0.216 |
| LZ3 | -0.378 | 0.178 | **0.045** | -1.067 | 0.387 | **0.011** | -0.600 | 0.298 | 0.056 | -2.045 | 0.751 | **0.012** | -0.316 | 0.398 | 0.435 |
| NHC | -0.009 | 0.010 | 0.366 | 0.015 | 0.022 | 0.511 | -0.018 | 0.016 | 0.281 | -0.012 | 0.044 | 0.792 | -0.010 | 0.020 | 0.617 |
| NTG | 0.003 | 0.002 | 0.190 | 0.007 | 0.004 | 0.110 | 0.005 | 0.003 | 0.142 | 0.015 | 0.008 | 0.093 | 0.002 | 0.004 | 0.574 |
| NVCTG | 0.002 | 0.002 | 0.233 | 0.007 | 0.004 | 0.114 | 0.004 | 0.003 | 0.200 | 0.014 | 0.008 | 0.116 | 0.002 | 0.004 | 0.659 |
| Platelets | -0.002 | 0.002 | 0.297 | -0.010 | 0.004 | **0.013** | -0.005 | 0.003 | 0.128 | -0.017 | 0.008 | **0.039** | -0.009 | 0.003 | **0.015** |
| TG | 0.003 | 0.002 | 0.108 | 0.008 | 0.003 | **0.042** | 0.005 | 0.002 | 0.058 | 0.015 | 0.007 | **0.036** | 0.003 | 0.003 | 0.414 |
| TG/HDLC | 0.053 | 0.063 | 0.408 | 0.156 | 0.150 | 0.310 | 0.227 | 0.097 | **0.031** | 0.437 | 0.284 | 0.141 | 0.090 | 0.133 | 0.507 |
| VLCP | 0.046 | 0.018 | **0.021** | 0.113 | 0.041 | **0.012** | 0.068 | 0.031 | **0.041** | 0.226 | 0.079 | **0.009** | 0.037 | 0.042 | 0.389 |
| VLDLCP | -0.002 | 0.004 | 0.571 | -0.003 | 0.008 | 0.729 | -0.003 | 0.006 | 0.627 | -0.008 | 0.016 | 0.625 | 0.001 | 0.008 | 0.851 |
| VMP | -0.003 | 0.006 | 0.625 | 0.000 | 0.014 | 0.980 | 0.003 | 0.010 | 0.801 | -0.001 | 0.028 | 0.977 | 0.004 | 0.013 | 0.742 |
| VSP | -0.008 | 0.006 | 0.231 | -0.020 | 0.014 | 0.177 | -0.019 | 0.010 | 0.073 | -0.046 | 0.027 | 0.100 | -0.004 | 0.013 | 0.779 |
| VZ | 0.040 | 0.011 | **0.001** | 0.080 | 0.026 | **0.006** | 0.056 | 0.019 | **0.008** | 0.177 | 0.048 | **0.001** | 0.016 | 0.028 | 0.568 |

Beta, standard error (S.E.) and significance (P value) revealed upon linear regression analysis of the indicated variable with individual components of NAFLD severity (inflammation, fat, ballooning), NAFLD Activity Score (NAS), and Fibrosis score. Values highlighted in red are *P* < 0.05.
